# Armillaria Root-Rot Pathogens: Species Boundaries and Global Distribution

**DOI:** 10.3390/pathogens7040083

**Published:** 2018-10-24

**Authors:** Martin P.A. Coetzee, Brenda D. Wingfield, Michael J. Wingfield

**Affiliations:** Department of Biochemistry, Genetics and Microbiology (BGM), Forestry and Agricultural Biotechnology Institute (FABI), University of Pretoria, Pretoria 0002, South Africa; brenda.wingfield@fabi.up.ac.za (B.D.W.); mike.wingfield@fabi.up.ac.za (M.J.W.)

**Keywords:** Basidiomycota, phylogenetics, fungal tree pathogens, fungal systematics, fungal biogeography

## Abstract

This review considers current knowledge surrounding species boundaries of the Armillaria root-rot pathogens and their distribution. In addition, a phylogenetic tree using translation elongation factor subunit 1-alpha (*tef*-1α) from isolates across the globe are used to present a global phylogenetic framework for the genus. Defining species boundaries based on DNA sequence-inferred phylogenies has been a central focus of contemporary mycology. The results of such studies have in many cases resolved the biogeographic history of species, mechanisms involved in dispersal, the taxonomy of species and how certain phenotypic characteristics have evolved throughout lineage diversification. Such advances have also occurred in the case of *Armillaria* spp. that include important causal agents of tree root rots. This commenced with the first phylogeny for *Armillaria* that was based on IGS-1 (intergenic spacer region one) DNA sequence data, published in 1992. Since then phylogenies were produced using alternative loci, either as single gene phylogenies or based on concatenated data. Collectively these phylogenies revealed species clusters in *Armillaria* linked to their geographic distributions and importantly species complexes that warrant further research.

## 1. Introduction

*Armillaria* spp. (Basidiomycota, Agaricales, Physalacriaceae) are amongst the best known and most important pathogens of forest trees but are also beneficial to horticulture and growth of edible fungi. They are found on almost every continent, have resulted in serious losses in sustainably managed natural forests as well as in plantations of non-native tree species [1]. Some species are important mycorrhizal symbionts of orchids [2,3,4,5,6]. Other species are symbionts of edible fungi such as *Polyporus umbellatus* and necessary for their production [7,8,9,10]. Most species have a saprophytic lifestyle during which they contribute to the decomposition of organic material in forest ecosystems and they become pathogens when environmental conditions are favourable for infection. *Armillaria* spp. have also become notoriously interesting to biologists associated with the fact that they include some of the world’s largest and oldest organisms [11,12].

The genus *Armillaria* has a long and controversial taxonomic history. This dates back to the 1700’s and the description of *Agaricus melleus* Vahl, Fl. dan.: t. (now *A. mellea*) by Danish Botanist Martin Vahl [13]. Subsequently, a number of taxonomic revisions were published and the valid name for the genus was often in dispute [14]. *Armillaria* Fr. Staude is now the broadly accepted generic name with *Armillaria mellea* (Vahl) P. Kumm. representing the type of the genus [14,15] (see [1] for characteristics of the genus). At least 129 species are currently recognised in *Armillaria* (MycoBank: html://http://www.mycobank.org/). In addition to these species, a number of biological species and phylogenetic groups have also been identified but these have yet to be formally described (e.g., [6]).

Phylogenetic studies have revolutionised our understanding of species boundaries in *Armillaria*. The first study to apply DNA sequence data to this question was conducted by Anderson and Stasovski [16]. This resulted in a phylogenetic concept for the relationships among *Armillaria* species from North America. This ground-breaking work was followed by a number of studies respectively dealing with species from Africa [17], Europe [18,19], Australasia [20,21], South America [22,23] and South-east Asia [6,24,25]. 

There have been few studies that have considered the phylogenetic relationships of *Armillaria* spp. at a global scale [26,27,28,29]. Maphosa et al. [26] and later Coetzee et al. [27] showed that that species of *Armillaria* form three distinct geographical clades. These include the African clade, the Holarctic clade (*sensu* [30]) and a non-Holarctic clade including several regions in the Holotropical and Austral kingdoms as defined by Morrone [30]. This subdivision was supported more recently by Koch et al. [29]. Using a concatenated dataset comprising six gene regions (rDNA large subunit [LSU], translation elongation factor subunit 1-alpha [*tef*-1α], RNA polymerase subunit II [*rpb2*], β-tubulin [TUB], Glyceraldehyde-3-phosphate dehydrogenase [*gpd*] and *actin-1*), the phylogenetic tree generated by these authors grouped *Armillaria* species from Africa, the Northern Hemisphere and Australian/temperate South America into clades representing those regions. In contrast to the results of Maphosa et al. [26] and Coetzee et al. [27], isolates of *A. mellea* formed a clade sister to the northern hemisphere—Australasian—temperate South America clades and the exannulated species (*A. ectypa* and *A. tabescens*) formed a monophyletic group basal to all *Armillaria* species. The placement of *A. ectypa* and *A. tabescens* basal to other *Armillaria* spp. and the fact that they are the only exannulated species provided sufficient support for the description of the new genus *Desarmillaria* [29]. Following the work of Koch et al. [29], Klopfenstein et al. [28] identified “superclades” in a global phylogeny of *Armillaria* spp. These were referred to as the Gallica, Solidipes/ostoyae, Mellea and Socialis/tabescens superclades.

The age of the most recent ancestor of *Armillaria* (including *Desarmillaria*) was dated within the Early Paleogene. Coetzee et al. [27] estimated the most recent ancestor of *Armilllaria* at 54 million years ago (MYA). This was supported by Koch et al. [29] who dated the most common ancestor of *Armillaria* at 41 MYA. Consequently, the age of the ancestral lineage of *Armillaria* post-dates the continental break up of Gondwana. This suggests that extant species attained their current biogeographical distribution through long-distance dispersal rather than by vicariance events as was previously hypothesised by [31].

This review considers species boundaries of the Armillaria root-rot pathogens and their distribution. For this purpose, we interrogated current knowledge regarding species clusters and phylogenetic relationships of species or taxa (biological species or lineages) based on morphological cohesion, phenotypic characteristics and phylogenetic analyses. A phylogenetic tree based on *tef*-1α DNA sequences was constructed using the dataset from Klopfenstein et al. [28] with additional sequences of taxa not included in their study and serves as phylogenetic framework for *Armillaria* in this review. A curated DNA sequence database was also constructed to aid in future studies.

## 2. Genes and Genomic Regions Employed in Phylogenetic Studies of *Armillaria* Species

Several genes and genomic regions have been employed in phylogenetic studies of *Armillaria* species (see Table S1 for a summary). Most early phylogenetic studies on *Armillaria* have incorporated the non-coding regions of the nuclear rRNA cistron to construct phylogenetic trees. Among these regions, the intergenic spacer 1 region (IGS-1), located between the nuclear ribosomal large subunit (nLSU, 28S gene) and the 5S gene, was first used to determine the relationships among North America *Armillaria* species [16]. In subsequent studies, the internally transcribed spacer regions (ITS-1 and ITS-2) together with the 5.8S gene (situated between the ITS-1 and ITS-2) were employed for phylogenetic inference [18,20,21]. Although these regions were successfully used in various studies, it was recognised that they fail to provide sufficient resolution to differentiate certain *Armillaria* species. Moreover, intra-strain nucleotide heterogeneity was reported for the ITS and IGS-1 regions in some species [32,33], making it impossible to sequence these regions without cloning. An additional complication was the fact that the 5S gene is inverted for the African *Armillaria* species relative to other species [17,34]. Consequently, the IGS-1 region cannot be used in a global phylogeny of *Armillaria* species. Thus, alternative loci were considered for phylogenetic purposes.

The protein-coding genes, or regions of these genes, most commonly used in the fungal phylogenetic analyses include the *tef*-1α and *rpb2* genes (see [35] for a detailed account). Following the study on *Phellinus nigrolimitatus* by Kauserud et al. [36] and using the primers developed by these authors, Maphosa et al. [26] published the first phylogeny of *Armillaria* species based on sequences from a region of the *tef*-1α gene. Baumgartner et al. [37] showed that *tef*-1α occurs as a single copy gene in *A. mellea*. However, Guo et al. [6] discovered some diploid strains of *Armillaria* that contained intra-strain heterozygous sites in the *tef*-1α region and suggested this might be due to hybridisation events. Despite this, several studies have shown that this region in general provides the necessary resolution to infer species relationships with confidence, especially if phylogenies of species from the same geographic region are considered [6,19,25,26,28,38,39,40].

Matheny et al. [35] showed that *rpb2* sequences can be used to resolve the phylogeny between closely related species in the Basidiomycota. This led to the research of Brazee et al. [39] to evaluate partial *tef*-1α, *rpb2*, and nLSU sequences for the identification of isolates representing *A. calvescens* and *A. gallica* from north-eastern North America and the application of this gene in a phylogeny of six *Armillaria* species from that geographic region. *Armillaria calvescens* and *A. gallica* are morphologically very similar with some differences in microscopic characters of their basidiocarps [41]. Brazee et al. [39] showed that sequences from the *rpb2* gene cannot discriminate between *A. calvescens* and *A. gallica*. However, they found that these species and the four other species included in their study (*A. gemina*, *A. mellea*, *A. sinapina*, and *A. solidipes*) could be separated based on *tef*-1α gene sequences. 

Recently, Guo et al. [6] utilised β–tubulin, in addition to ITS and *tef*-1α, sequence to study the phylogenetic relationships of Chinese biological species of *Armillaria*. β–tubulin and *tef*-1α gene trees generated in that study conflicted in the monophyly of strains. Strains that formed a monophyletic group in the *tef*-1α gene tree formed sub-clades distant to each other in the β–tubulin gene tree. β–tubulin genes underwent multiple independent duplications and losses in fungal lineages [42]. It is thus possible that paralogous copies were retained in different strains of *Armillaria* yielding paraphyletic groups in phylogenetic analyses. Sequences for β–tubulin have consequently not been used in more recent phylogenetic studies and it is doubtful if the gene carries adequate phylogenetic signal to provide a robust phylogeny for *Armillaria* species.

In addition to the loci described above, anonymous sequences generated through Sequencing With Arbitrary Primer Pairs (SWAPP) were used to determine the phylogeny of North American biological species [43]. Phylogenetic analysis of a matrix in which these sequences were combined yielded a phylogenetic tree that resolved the phylogenetic history of the North American species [43]. This method, however, has not been employed in subsequent phylogenetic studies of *Armillaria*.

## 3. Curation of Sequences from GenBank and Phylogenetic Analyses

A large number of DNA sequences for *Armillaria* species are available in GenBank. As is the case with many other fungal sequence deposits, many of these are not linked to published papers. The authenticity and origin of these sequences are often questionable. Another problem is that strain numbers can be linked to deposited sequences where the same strain has been assigned different strain numbers by researchers in different laboratories. Utilisation of these data can consequently lead to an artificial inflation of the taxon sampling in phylogenetic studies.

A database that curates high quality published sequence data linked to strain and the biogeographic information is currently not available. Therefore, we obtained sequences for the ITS, IGS-1, *tef*-1α and LSU regions from GenBank using published accession numbers rather than blast or keyword searches. A “bionumber” was assigned that linked individual strains to their available DNA sequences, their different strain numbers published by different researchers, biogeographic information and publication references. This information is available in a Microsoft^®^ Excel Spreadsheet accessible at: http://www.fabinet.up.ac.za/mcoetzee (Table S2) and is updated when sequences in new publications become available.

**Figure 1:**
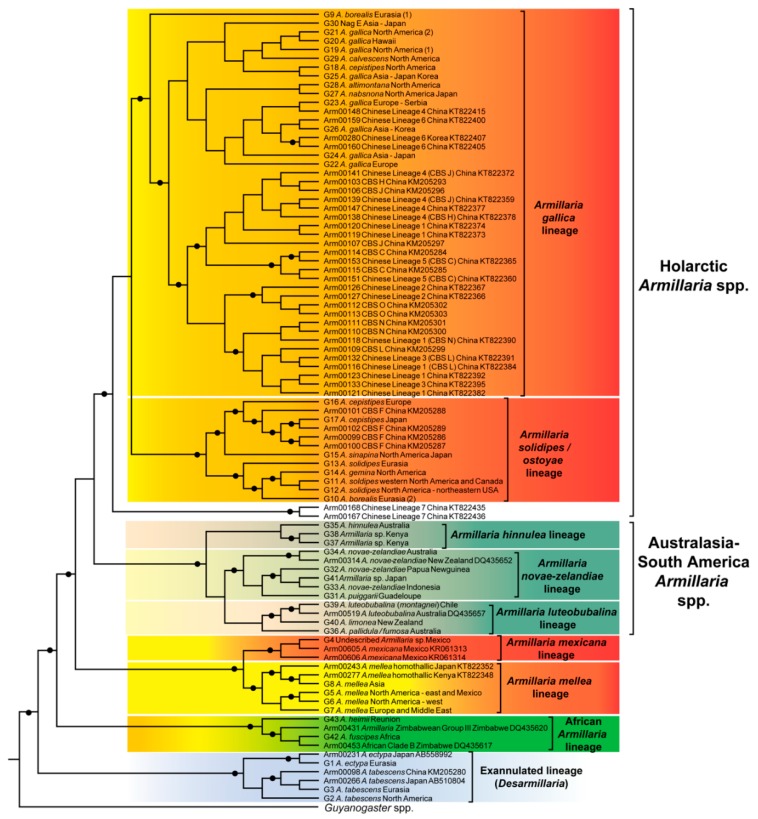
Phylogenetic tree generated from *tef*-1α DNA sequence data showing the phylogenetic relationships of *Armillaria* species and lineages. Circles at nodes indicate posterior probability values > 0.95. Group numbers from Klopfenstein et al. [28] are shown at the species names where applicable. Arm numbers are bionumbers link to published sequences of *Armillaria* strains and for which information is provided in Table S2.

A phylogenetic tree based on *tef*-1α sequence data and trees based on published phylogenies was generated for this review (Figure 1 and Figure S1). The *tef*-1α data set comprised the sequence dataset of Klopfenstein et al. [28] and additional sequences obtained from GenBank (Table S3). In total the dataset included 94 taxa, of which 47 *Armillaria* sequences were new additions. Sequences were re-aligned using the online version of MAFFT version 7 [44] with default settings. The phylogenetic tree was constructed based on Bayesian inference of phylogenies using BEAST version 1.10 [45] and following the protocol described in Klopfenstein et al. [28]. *Guyanagaster necrorhizus* was used as the outgroup species to root the tree. Published phylogenetic trees were redrawn because *tef*-1α sequence data were not available for all taxa or species. This also made it possible to indicate alternative phylogenetic positions of taxa or species where loci other than *tef*-1α were used for phylogenetic analyses. For this purpose, manually edited trees were generated in Mesquite version 3.5 [46] with the phylogenetic position of taxa based on their published relationship with other *Armillaria* species (Figure S1).

## 4. Relatives of *Armillaria*

*Armillaria* was originally assigned to the Tricholomataceae, one of the largest families in the Agaricales that included a diverse assemblage of genera. Molecular systematic studies revealed the polyphyly of the family [47,48,49,50]. Several genera previously residing in the Tricholomataceae were transferred to other families by Matheny et al. [49] based on their monophyletic groupings. In that study, *Armillaria* formed a monophyletic group together with other genera that were assigned to the Physalacriaceae. This family together with several other families that also include the Marasmiaceae constitute the Marasimioid clade in the Agaricales [49].

The question regarding the closest relative to *Armillaria* is intriguing. Currently, the phylogenetically closest known relatives of *Armillaria* are *Guyanagaster necrorhizus* and *G. lucianii* [29,51], sequestrate fungi that are known only from the neotropical rainforests of the Guiana Shield (Pakaraima Mountains, Guyana). Phylogenetic analyses that included various genera in the Agaricales and using DNA sequences for five loci (18S, ITS, and 28S rDNA, *rpb2*, and *tef*-1α) placed *G. necrorhizus* sister to *A. tabescens* and *A. mellea* [51]. Subsequent to this study, Koch et al. [29] inferred the phylogenetic relationship of *Guyanagaster* relative to the global collection of *Armillaria* species. That study showed that *G. necrorhizus* and *G. lucianii* is a monophyletic group sister to *Armillaria* and the newly erected genus *Desarmillaria*.

For many years it was suggested that *A. tabescens*, which was referred to as *A. socialis* in Europe [52], and *A. ectypa* should be moved to discrete genera [52,53]. This notion was based on the fact that these are the only known *Armillaria* species that do not produce an annulus on the stipe at maturity. It was further thought that *A. socialis* (*A. tabescens*) is more thermophilic than other *Armillaria* species residing in the Northern Hemisphere [52,54]. Pegler [53] treated *A. tabescens* and *A. ectypa* in the section *Desarmillaria*, but without making a formal transfer to that genus. 

The placement of *A. ectypa* and *A. tabescens* in a genus different to *Armillaria* is contentious because both genera have morphological features typical of *Armillaria* spp., including basidiospore morphology, the production of rhizomorphs in culture and diploid secondary mycelium. Furthermore, phylogenetic trees generated by Maphosa et al. [26] and Coetzee et al. [27] (Figure S1a) clustered the exannulated species in a monophyletic group that included *Armillaria* species from the Northern Hemisphere. Recently, phylogenetic analysis (Figure S1b) based on six gene regions by Koch et al. [29], however, showed that *A. tabescens* and *A. ectypa* reside in a monophyletic group sister to a clade that includes all known *Armillaria* lineages. Based on their morphological differences and phylogenetic position relative to other *Armillaria* spp. the authors formally erected the genus *Desarmillaria* and introduced the new combinations *D. tabescens* and *D. ectypa* to accommodate the two exannulated species of “*Armillaria*”. *Desarmillaria* consequently represents the phylogenetically closest known genus to *Armillaria.* In the present review, *A. tabescens* and *A. ectypa* are treated as *Armillaria* due to their taxonomic history and similarity to other *Armillaria* species.

## 5. Species Clusters and Phylogenetic Relationships Based on Morphological Cohesion, Phenotypic Characteristics and Phylogenetic Analyses

Most early studies on *Armillaria* focused on relationships among those species residing in the Holarctic region. But more recently, there has been work on species from the non-Holarctic regions, mainly focusing on species from sub-Sahara Africa, Australia and New Zealand [17,20,21]. It was only relatively recently that efforts were made to resolve phylogenetic relationships among species in South America [23]. Collectively, these studies suggest that *Armillaria* species reside in clusters, which we refer to here as lineages, that reflect their origin, morphological similarity and ecological characteristics. It has also been shown that the Australasian-South American species form a monophyletic group sister to the Holarctic species (with the exception of *A. mellea* and *A. mexicana*) and that species from Africa are a basal group in *Armillaria* as illustrated in the present study and supported by [28,29]. These different lineages are discussed below.

### 5.1. Species Lineages from the Holarctic

In this review, five Holarctic lineages are recognised (Figure 1). These are referred to as the *A. gallica, A. solidipes*/*ostoyae*, *A. mellea*, *A. mexicana* and Exannulated lineages based on results from Coetzee et al. [27] and Klopfenstein et al. [28] (although the latter publication referred to these lineages as superclades) as well as the analysis conducted for this review. *Armillaria tabescens* and *A. ectypa* are collectively referred to in the present review as the Exannulated lineage. Results of phylogenetic analyses by Coetzee et al. [27] and Klopfenstein et al. [28] suggested that the *Armillaria gallica* lineage forms a sister group with the *A. solidipes*/*ostoyae* lineage. The phylogeny produced by Coetzee et al. [27] indicated that the *A. mellea* and the Exannulated lineages are basal to the Holarctic species. However, this was not supported in the phylogenetic trees of Klopfenstein et al. [28], Koch et al. [29] and neither in the phylogenetic analyses emerging from the present study. Rather, the remainder of the Northern Hemisphere lineages were shown to be more closely related to lineages from the Southern Hemisphere than to the *A. mellea* and Exannulated lineages. An in-depth discussion dealing with the northern hemisphere lineages is provided in Klopfenstein et al. [28], therefore only the main issues relating the phylogeny and taxonomy of species in these lineages are provided below.

#### 5.1.1. The *Armillaria solidipes/ostoyae* Lineage

**Figure 2:**
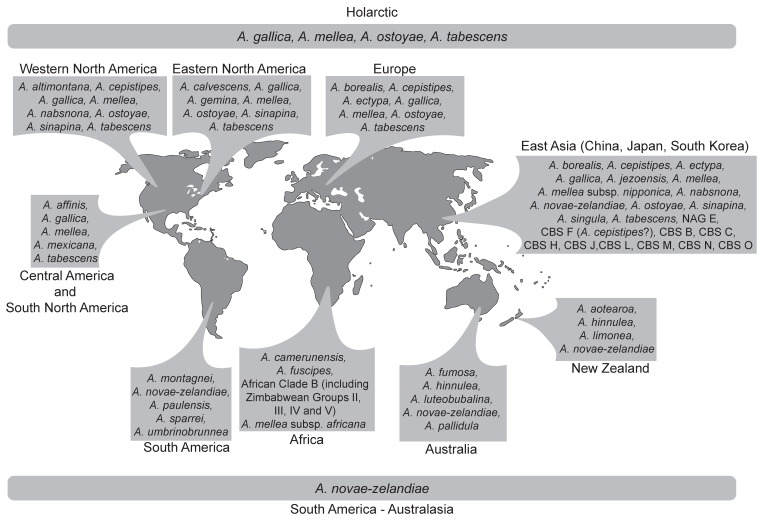
Map showing the geographic distribution of *Armillaria* species and biological species not assigned to morphological species.

Species in the *Armillaria solidipes*/*ostoyae* lineage include: *A. ostoyae*, *A. borealis* and *A. gemina*, *A. sinapina* (G15, North America and Japan) and *A. cepistipes* (G16 from Europe and G17 from Japan) and Chinese Biological Species (CBS) F (proposed as being *A. cepistipes* by [6]) based on phylogenetic trees (Figure 1) generated from *tef*-1α DNA sequence data [25,28]. Within this group, *A. ostoyae*, *A. borealis* and *A. gemina*, are morphologically similar in having a thick anulus, stipes that are more or less equal in shape and the presence of distinct dark scales [55]. *Armillaria ostoyae* (proposed by [56] as *A. solidipes* because this is the older name, but not always accepted [57]) has a transcontinental distribution, occurring in Europe, North America and Asia (China, Japan and South Korea), *A. borealis* occurs in both Europe and Asia (China) while *A. gemina* is confined to north-eastern North America [31,58,59,60] and as shown in Figure 2 (Table S4).

Phylogenetic studies have revealed that *A. ostoyae*, *A. borealis* and *A. gemina* are phylogenetically more closely related to each other than they are to the other *Armillaria* species from the Holarctic region (Figure 1). Anderson et al. [61] showed, using rDNA Restriction Fragment Length Polymorphism (RFLP) profiles, that the three species harbour distinct rDNA classes. This was later reflected in their grouping based on ITS and IGS-1 sequence phylogenies that separated them from other Holarctic *Armillaria* species [16,18]. More recent research has, however, shown that strains representing *A. borealis* and *A. ostoyae*, based on their identification using mating tests, have polyphyletic origins [6,19,25,28,62]. Strains of *A. ostoyae* reside in two paraphyletic lineages based on IGS-1 and *tef*-1α sequences (Figure 1), with one lineage grouping within a monophyletic group with *A. borealis* and *A. gemina* [25,28]. IGS-1 sequences group strains of *A. borealis* in a monophyletic group [25], however, phylogenetic trees generated from *tef*-1α gene sequences (Figure 1) place them in paraphyletic groups [25,28]. One group forms a monophyletic group with *A. gemina* and *A. ostoyae*, while strains from the second group cluster in a monophyletic group distant to the *A. solidipes/ostoyae* lineage. Overall the results of these studies suggest that *A. ostoyae* and *A. borealis* represent species complexes with cryptic species that cannot be detected using mating tests.

Phylogenetic studies based on ITS, IGS-1 or a combination of these regions together with *tef*-1α sequences have shown that isolates of *A. sinapina* and *A. cepistipes* are closely related to *Armillaria* species in the *A. gallica* lineage [25,63,64]. However, phylogenetic trees generated by different authors based on *tef*-1α sequence data alone suggested that some isolates representing these species are closely related to *A. borealis*, *A. gemina* and *A. ostoyae* [6,64]. Most recently, Klopfenstein et al. [28] showed that *A. sinapina* (Group 15, from North America and Japan) and two groups of *A. cepistipes* (Group 16 from Europe and Group 17 from Japan) constitute a monophyletic group sister to *A. borealis*—*A. solidipes*/*ostoyae*—*A. gemina* with strong statistical support. This was also the case in the current analysis (Figure 1).

#### 5.1.2. The *Armillaria gallica* Lineage

The *Armillaria gallica* lineage represents the largest and most complex group of Holarctic species. The cluster includes *A. altimontana*, *A. calvescens*, *A. cepistipes*, *A. gallica*, *A. jezoensis*, *A. nabsnona*, *A. sinapina* and *A. singula* (Figure 1 and Figure 2, Table S4). Several biological species and phylogenetic lineages that are not yet linked to morphological species have been identified and reside in this lineage (Figure 1, Table S4). Species in this lineage can generally be distinguished by their thin delicate annuli and more bulbose or clavate stipes [55]. The species residing in this cluster differ in their geographical distributions, some being confined to certain continents while others have a transcontinental distribution (Figure 2, Table S4).

Determining the phylogenetic position of species in the *A. gallica* lineage is complicated by gene trees in which isolates of the same species occur at different positions, isolates supposed to represent the same species being placed distant to each other in phylogenetic trees generated from the same locus and overall low statistical support for nodes [25,28]. The taxonomic status of *A. calvescens*, *A. nabsnona*, *A. altimontana* and NAG E is supported by the results of several studies that showed that isolates representing these species formed distinct monophyletic clades [6,25,28] (Figure 1). However, their phylogenetic position relative to each other and the remaining *Armillaria* species in this cluster is not clear due to low statistical support in the published phylogenetic trees [6,25,28] and in our analyses for this review.

Within the *A. gallica* lineage, the taxonomy of *A. gallica* and *A. cepistipes* is problematic since phylogenetic studies have shown that these species are polyphyletic [6,25,28]. DNA sequence analyses revealed that European and Northern American *A. gallica* are phylogenetically distinct [62] and that *A. gallica* from East-Asia is genetically different to European *A. gallica* [6]. It is also known that *A. gallica* constitutes up to eight clades [28] with multiple phylogenetic groups being present in North America [65], Europe [66] and Asia [6,25]. Guo et al. [6] suggested that *A. gallica* is restricted to Europe and “*A. gallica*” from East Asia and North America should be treated as a different species. *Armillaria cepistipes* from North America resides in the *A. gallica* lineage while *A. cepistipes* from Europe and Japan cluster with species in the *A. solidipes*/*ostoyae* lineage based on *tef*-1α sequence data [25,28] (Figure 1). But, phylogenetic trees based on sequences for other loci group *A. cepistipes* from the different regions in the *A. gallica* lineage [25,38,63]. Collectively these studies have shown that *A. gallica* and *A. cepistipes* represent species complexes with several cryptic species that warrant further taxonomic investigation.

Several biological species that are restricted to China (Figure 2, Table S4) and that were later assigned to phylogenetic lineages [6] reside in the *A. gallica* lineage [25]. The Chinese biological species (CBS) in this lineage include CBS C, J, H, O, M and L (Figure 1). The phylogenetic relationships among these biological species are presently not resolved because the loci used in phylogenetic analyses do not provide sufficient phylogenetic signal for a robust phylogeny [6,25]. This is further complicated by the fact that single gene genealogies or concatenated data yield phylogenetic trees in which a phylogenetic lineage includes more than one biological species, while isolates identified as being the same biological species may reside in more than one phylogenetic lineage [6] (Table S4). 

#### 5.1.3. The *Armillaria mellea* Lineage

*Armillaria mellea* is the only species accommodated in this lineage (Figure 1). Representatives of this lineage are characterised by the lack of clamp connections at the base of their basidia, the presence of a distinct annulus, honey coloured caps and robust basidiocarps [67]. Isolates in the lineage have a transcontinental distribution occurring in eastern, western and central North America [31,65,68,69], Europe [58], Middle East [70] and Asia [59,71] (Figure 2). *Armillaria mellea* is also known from South Africa [72], Tanzania, Ethiopia, Sao Tome and Kenya [73,74,75] and as illustrated in Figure 2 (Table S4). However, these isolates represent examples of accidental introductions into new environments [72,76,77,78].

Members of the *A. mellea* lineage display considerable intraspecific variation at the DNA sequence level. Phylogenetic studies based on ITS, IGS-1 and *tef*-1α sequence data have revealed geographic clades within the lineage, distinguished by their origins. These include clades from Europe, western and eastern North America and Asia [26,79]. Population differentiation between western and eastern North America was later supported by microsatellite analysis [69]. The phylogenetic relationships among the clades are, however, not clear since the phylogenetic position of the clades based on different loci are not congruent. In view of the intraspecific variation in *A. mellea*, it was suggested that geographically separated populations of this species are in the process of speciation as a result of genetic isolation [79]. Based on DNA sequence variation between the geographical clusters, we hypothesise that the *A. mellea* lineage represents a species complex that is comprised of different cryptic species.

Members of the *A. mellea* lineage display intraspecific variation in their sexual systems. Similar to most *Armillaria* species, isolates that have been identified as *A. mellea* from Europe and North America and the majority of isolates from China are heterothallic. Homothallic forms were reported from Japan and China [59,80] as well as from the African countries of Ethiopia, Kenya, Tanzania, and Sao Tome [75,81]. The homothallic mating system was shown to be a unique form of secondary homothallism, and is therefore referred to as non-heterothallic [80]. The non-heterothallic isolates of *A. mellea* from Africa and Japan have been recognised as *A. mellea* ssp. *africana* [31] and *A. mellea* ssp. *nipponica* [82], respectively. Ota et al. [81] found that the Japanese population has high genetic diversity while African *A. mellea* isolates were identical to Japanese somatic incompatibility group A isolates based on somatic compatibility tests, isozyme and Random Amplification of Polymorphic DNA (RAPD) analyses. The authors therefore hypothesised that *A. mellea* spp. *africana* originated from East Asia. Phylogenetic analyses of sequences for *tef*-1α and ITS showed that the non-heterothallic and heterothallic forms from Asia and Africa are closely related in a clade [6,25]. Consequently, they cannot be differentiated based on the currently available DNA sequence data. However, Ota et al. [81] separated the two mating forms based on RAPDs, but isolates from China were not included (only isolates from Japan) in their study.

#### 5.1.4. The *Armillaria mexicana* Lineage

*Armillaria mexicana* is the only species accommodated in this lineage (Figure 1) and is known only from Mexico (Figure 2, Table S4). The species was placed with *A. mellea* in the “Mellea superclade” as denoted by Klopfenstein et al. [28]. However, with the exception of fibulae that are absent in the basidiomata, this species shows no other morphological similarity with *A. mellea* [83]. Isolates representing this species form a monophyletic group sister to *A. mellea* [28,83] (Figure 1). Furthermore, this species is unique in having the largest ITS region thus far reported for fungi with an ITS1 of 1299 bp and an ITS2 of 582 bp [83]. By virtue of these characteristics, *A. mexicana* is considered to represent a well-defined lineage in this review.

#### 5.1.5. The Exannulated Lineage of Species

*Armillaria tabescens* (*A. socialis* [52]) and *A. ectypa* (now referred to as *Desarmillaria tabescens* and *D. ectypa* [29]) are characterised by the lack of an annulus on their stipes. The two species differ in terms of their ecology and distribution (Table S4). *Armillaria ectypa* has a narrow distribution, being reported only from Europe (see [84] for an account of the distribution and references), Turkey [85], China ([86] cited in [59]) and Japan [87,88]. In Europe it is an extremely rare species and in many countries is considered to be endangered. *Armillaria ectypa* occurs as a saprophyte on woody material found in peat bogs and marshy habitats (e.g., [54]). *Armillaria tabescens* has a much broader distribution than *A. ectypa* being reported from southern Europe [52,58], North and Central America [89,90,91], South Korea [60,92,93], southern Japan [71] and China [59,94]. In some of these regions they are considered important pathogens on woody shrubs and trees (e.g., [91]).

Phylogenetic trees generated from different loci have suggested that *A. ectypa* and *A. tabescens* speciated from a common ancestor. In a study by Ota et al. [95], isolates representing *A. ectypa* and *A. tabescens*, respectively, formed sister groups. However, this relationship did not receive statistical support through bootstrap analysis and an outgroup to polarise the tree was not included in their study. The sister relationship between *A. ectypa* and *A. tabescens* was strongly supported in the phylogenetic trees presented in the studies of Coetzee et al. [27], Koch et al. [29] and Klopfenstein et al. [28].

The taxonomy of *A. tabescens* from North America, Europe and Asia is controversial due to inconclusive results emerging from sexual compatibility and phylogenetic studies. Sexual compatibility between isolates from North America and one isolate from Italy suggested that these isolates represent the same species [96]. In contrast, Guillaumin et al. [58] reported that *A. tabescens* from Europe is compatible with isolates from Asia but not with isolates from North America. Kile et al. [31] consequently suggested that *A. tabescens* from North America should be assigned to *A. monodelpha,* but this name was considered illegitimate by Volk et al. [14]. Ota et al. [71] found that strains from Japan were interfertile with European strains but intersterile with one isolate from North America. Later, Qin et al. [59] reported that strains from China (CBS I) are sexually compatible with strains of *A. tabescens* from Europe. In a more recent phylogenetic study, Tsykun et al. [64] showed that European and Japanese isolates of *A. tabescens* form separate clades when sequences from the IGS-1, ITS, and *tef*-1α regions are combined. That study, however, included a limited number of samples of *A. tabescens* and the grouping of Japanese isolates received marginal statistical support.

In view of the discrepancies in sexual compatibility between isolates of *A. tabescens* from the different regions, Coetzee et al. [25] attempted to resolve the taxonomic uncertainty following a phylogenetic approach. Results of their study supported the notion that *A. tabescens* from North America, Europe and Asia should be treated as a single taxon. The analyses presented by Coetzee et al. [25] was based on sequences for only two loci that are linked (ITS and IGS-1) and limited strain sampling. There is consequently a need for further studies before a definitive conclusion can be reached regarding the taxonomic treatment of *A. tabescens* from Europe, North America and Asia.

### 5.2. Species Lineages from Australasia-South America

In comparison to the Holarctic species, studies focusing on the phylogenetic relationships of *Armillaria* species from the non-Holarctic regions, excluding sub-Sahara Africa, are limited in number. To date, the most comprehensive work has been conducted on species from Australia and New Zealand [20,21] and the Patagonian Andes [23]. Results of these studies showed that the Australasian-South American *Armillaria* species represent a very divergent group of fungi in terms of their DNA sequence characteristics. The IGS-1 ranged between 400 to 1500 bp among different species [20], in comparison to the narrow size range (845–920 bp) among the Holarctic species [64,66,97]. Intra-strain sequence heterogeneity of the IGS-1 copies [20] and heterogenic multi copies of the *tef*-1α gene within the genomes of certain species [23] have been reported. Due to this variation, phylogenetic studies focussed mainly on ITS and LSU sequence data to infer phylogenetic relationships. Collectively, results of these studies showed that the *Armillaria* species from South America and Australasia reside in clades that we refer to as the *Armillaria hinnulea* lineage, *Armillaria novae-zelandiae* lineage and *Armillaria luteobubalina* lineage.

#### 5.2.1. The *Armillaria hinnulea* Lineage

The *Armillaria hinnulea* lineage includes *A. hinnulea*, *A. aotearoa*, *A. umbrinobrunnea* and *A. sparrei* (Figure 1 and Figure S1e). *Armillaria hinnulea* is found in Tasmania, south-eastern Australia and *Nothofagus* forests in the north-western part of the South Island of New Zealand [98,99,100] (Figure 2, Table S4). *Armillaria aotearoa* has a restricted distribution known only from the North and South Islands of New Zealand and is morphologically similar to *A. hinnulea* ([100] and references therein). *Armillaria umbrinobrunnea* and *A. sparrei* are restricted to South America ([101] and references therein) (Figure 2, Table S4).

Phylogenetic inference based on ITS sequences has revealed that *A. hinnulea* and *A. aotearoa*, previously referred to as “unknown species” [20,26], are more closely related to *Armillaria* species from the Holarctic than to those from Australia, New Zealand and South America [20] (Figure S1d). However, phylogenetic trees generated from *tef*-1α sequences (Figure 1) placed *A. hinnulea* sister to *A. fumosa* and *A. pallidula* that resides in the *Armillaria novae-zelandiae* lineage [26]. It was suggested that the incongruence in the gene tree topologies was due to the rapid evolution of the ITS region and that the *tef*-1α gene of *A. hinnulea* retained the ancestral character states of species within the non-Holarctic group [26].

The close affinity of *A. hinnulea* with species from the Holarctic is supported by morphological characteristics. Similar to species in the *A. gallica* and *A. ostoyae* clusters, *A. hinnulea* produces clamp connections in sub-hymenial layers of the basidiocarp [98]. These findings are also consistent with the view of Kile and Watling [98] that *A. hinnulea* resembles the Holarctic species *A. cepistipes*.

#### 5.2.2. The *Armillaria novae-zelandiae* Lineage

The *Armillaria novae-zelandiae* lineage accommodates *A. novae-zelandiae* and one isolate of *A. puiggarii* from Guadeloupe (Figure 1). Among the species occurring in the South American and Australasia regions, *A. novae-zelandiae* has the widest geographic distribution, occurring in Australia, New Zealand, South America, Papua New Guinea, Indonesia, Malaysia and Amami-Oshima, which is a subtropical island of Japan [22,95,98,102] (Figure 2, Table S4).

Comparison of published phylogenetic trees and those emerging from the present study (Figure 1) suggest four separate lineages for isolates of *A. novae-zelandiae* [20,22,23,26,95,101]. These also reflect their geographic origins: Australia, New Zealand, South America (Argentina and Chile) and Asia (Indonesia, Malaysia and Amami-Oshimi). Isolates from Australia and New Zealand are reciprocally monophyletic but considered conspecific by virtue of their similar basidiocarp morphology, vegetative growth characteristics and sexual compatibility [98,103]. The South American lineage is sister to the Australasian clade [22,23], while isolates from Asia are a basal monophyletic lineage within *A. novae-zelandiae* [22,26,95]. Ota et al. [95] reported that the basidiocarp morphology of collections from Amami-Oshimi closely resembles that of *A. fuscipes* from Africa. However, it is phylogenetically distantly related to African species and could therefore represent a newly discovered species. The lineages within *A. novae-zelandiae* suggest that the separate populations are in the process of allopatric speciation due to geographic isolation.

#### 5.2.3. The *Armillaria luteobubalina* Lineage

The *Armillaria luteobubalina* lineage is represented by several species that have different distribution patterns in the southern hemisphere (Figure 1 and Figure 2, Table S4). This group includes *A. fumosa*, *A. pallidula*, *A. luteobubalina*, *A. limonea*, *A. montagnei* and *A. paulensis*. *Armillaria pallidula*, *A. fumosa* and *A. luteobubalina* are restricted to Australia [104]. *Armillaria montagnei* has been identified in Chile and Argentina [22,101]. *Armillaria limonea* was reported from New Zealand and South America (Chile and Argentina) [105,106,107]. *Armillaria paulensis* is known from only one location in the south of São Paulo City [108].

The relationships among species within the *A. luteobubalina* lineage are not yet clear. This is due to topological differences among single locus trees and lack of nodal support for certain species groupings. However, comparisons of published phylogenetic trees have revealed a clustering of certain species that are supported by phenotypic traits.

*Armillaria pallidula* and *A. fumosa* together with *A. luteobubalina* and *A. montagnei* are pairs of sibling species based on individual ITS, *tef*-1α and LSU phylogenetic trees [20,23,26] (Figure 1 and Figure S1a,e). *Armillaria pallidula* and *A. fumosa* cannot be differentiated based on ITS or *tef*-1α DNA sequences [20,26] and they are similar in some morphological features [104]. However, they were shown to be distinct biological species based on interfertility tests [104]. The sister relationship between *A. luteobubalina* and *A. montagnei* is supported by pileus characteristics and an apparently “unpleasant” flavour [101,109,110]. Phylogenetic trees generated from ITS sequence data showed that *A. paulensis* is a close evolutionary relative of the *A. luteobubalina*–*A. montagnei* group [23,108].

### 5.3. The African Armillaria Lineage

The African lineage includes two major clades [17,26,111], referred to as *A. fuscipes* (=*A. heimii*) and African Clade B [17]. A disguising feature of this cluster is the fact that the 5S gene is inverted relative to other *Armillaria* species [17,34]. In addition, all isolates included in the studies of several authors [75,112,113] lack a tetrapolar (bifactorial) heterothallic mating system with some being bipolar (unifactorial) heterothallic while others are homothallic.

A number of studies have revealed sub-groups within the two major African clades. Two sister sub-groups were recognised in the *A. fuscipes* clade represented by isolates from Ethiopia and Kenya, respectively, based on ITS and IGS-1 sequence data [17,114]. Clade B is the most diverse group based on ITS-1 and IGS-1 sequences as well as AFLP and somatic compatibility studies. Together with other isolates from Africa, this clade includes sub-groups referred to as Zimbabwean Groups II, III, IV and V [115,116]. Collectively, results of these studies suggest that the different sub-groups in the major clades possibly represent distinct species. But, Pérez-Sierra et al. [111] were of the view that the major clades and their sub-groups were a single species comprising different molecular groups.

In addition to the taxa described above and *A. mellea* ssp. *africana*, an additional lineage occurring on tea (*Camellia sinensis*) in Kenya has been recognised. The lineage is referred to as Kenyan Group III [117], SIG (somatic incompatibility group) II [118], SIG III [119] and Kenyan Group II [120] by different authors. Different from other African taxa, this group is more similar to northern hemisphere species in that the 5S gene is not inverted [117,120]. Furthermore, it is phylogenetically more closely related to northern hemisphere species [120], and formed a sister group with *A. hinnulea* based on IGS-1 [117] and *tef*-1α sequences [28] (Figure 1). We postulate that the fungus was most likely introduced into Africa.

## 6. Conclusions and Future Prospects

Substantial progress has been made during the past 26 years towards understanding the identity and phylogenetic relationships of Armillaria root pathogens. Using DNA sequence data and phylogenetic methods, new species are regularly being discovered or recognised. However, there is still much to be learned regarding the species boundaries between closely related taxa, especially those in the *A. gallica* lineage. But, questions relating to species boundaries of these taxa will be resolved only by molecular phylogenies based on DNA or amino acid sequences from multiple independent evolving loci.

Genealogical Concordance Phylogenetic Species Recognition (GCPSR) using the concordance among several gene trees [121,122] to delineate species has become standard in fungal taxonomy. But, with the exception of a few studies (e.g., [6,64]) this taxonomic method has not yet been widely implemented in *Armillaria* taxonomy. The limited application of GCPSR in *Armillaria* taxonomic studies can be ascribed mainly to the absence of a standard set of informative unlinked single copy genes. This warrants a concerted effort to identify an assemblage of genes that will facilitate GCPSR in *Armillaria* taxonomy.

Phylogenomics based on a large collection of orthologous genes or transcriptomes provides a promising method for future research that focuses on *Armillaria* species boundaries, constructing robust phylogenetic frameworks, and to diagnose species. Earlier work by Rokas et al. [123] showed that at least 20 unlinked genes or 8000 randomly selected orthologous nucleotides distributed over the sampled genomes are required to construct a robust species phylogeny and this will probably be the case for *Armillaria* phylogenomics. Using a phylogenomic approach has already lead to progress in fungal systematics and the diagnoses of species new to science [124]. It is evident that the reduction in cost associated with whole genomes sequencing [125], advances in whole genome sequence technology and bioinformatic pipelines will make it feasible for research groups to conduct phylogenomic studies on large collections of *Armillaria* isolates. Even if whole genomes cannot be sequenced, several genome sampling methods are available to obtain phylogenetic informative characters that can be used in phylogenomic studies at various scales (see [126]). Sequences of the genomes of key species [127] are already providing exciting prospects to study the evolution and systematics of *Armillaria*. They are certain to lead to important breakthroughs regarding not only the taxonomy but the biology and ecology of these fascinating fungi in the future. 

## Supplementary Materials

The following are available online at http://www.mdpi.com/2076-0817/7/4/83/s1. Table S1: Summary of genes and genomic regions employed in phylogenetic studies of *Armillaria* species. Table S2: Curated database of published DNA sequences from GenBank for *Armillaria* species. Table S3: Additional *tef*-1α DNA sequences from *Armillaria* species or taxa included in this study. Table S4: Distribution of *Armillaria* species and taxa from phylogenetic studies and their associated biological species designation. Figure S1. Phylogenetic trees generated from publications for species for which *tef*-1α DNA are not available or that had conflicting phylogenetic positions based on genomic regions other than *tef*-1α.
