# Supplementary material for: Armillaria Root-Rot Pathogens: Species Boundaries and Global Distribution
Source: Pathogens. 2018 Oct 24;7(4):83. doi: 10.3390/pathogens7040083 (PMC6313743; doi:10.3390/pathogens7040083)
Supplement: Supplementary file 1 [file pathogens-07-00083-s001.zip › pathogens-368392-supplementrary final/Figure S1 Published trees v2.pdf]

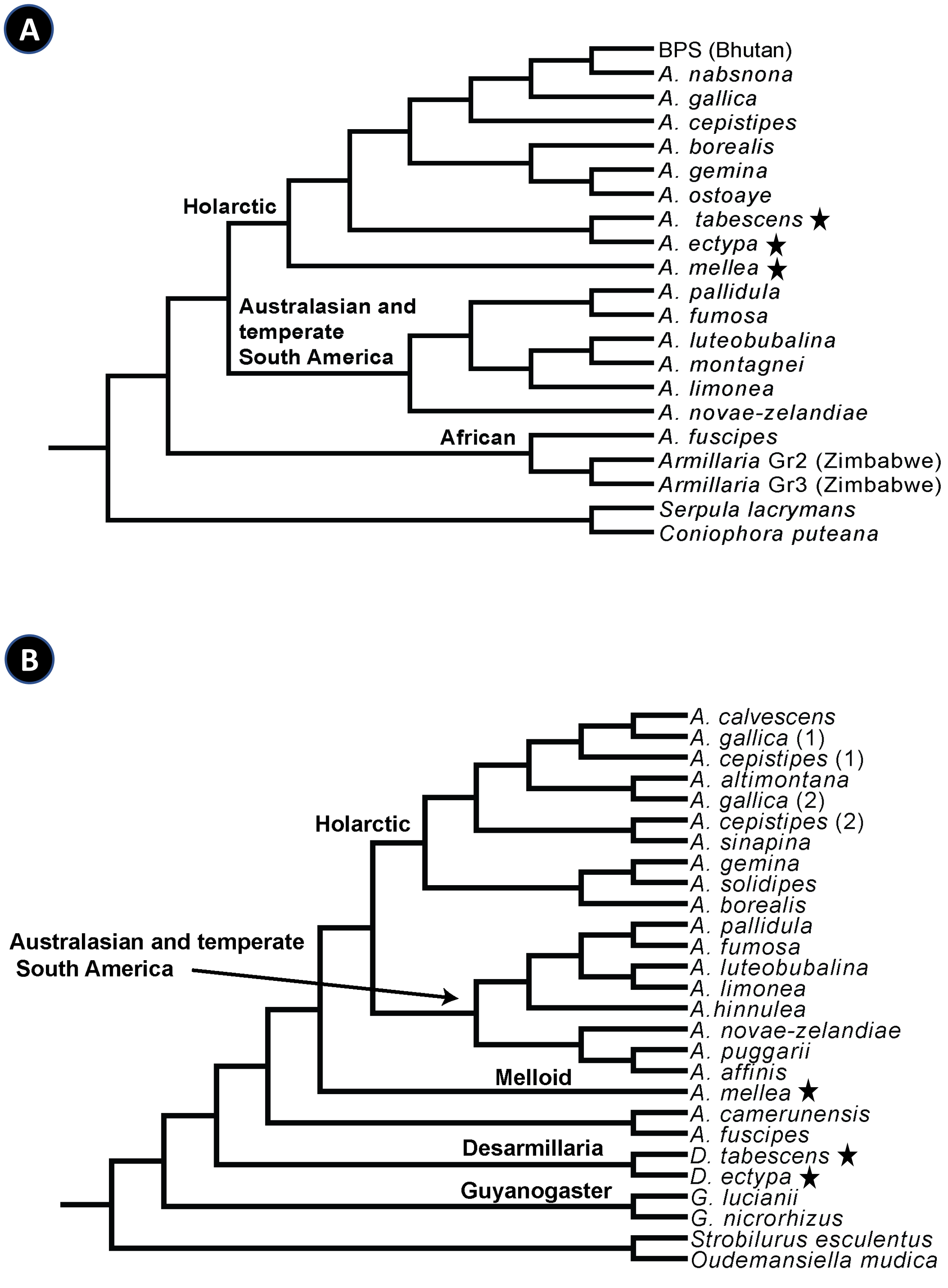

Figure S1. Phylogenetic trees generated from publications for species for which *tef*-1α DNA are not available or that had conflicting phylogenetic positions based on genomic regions other than *tef*-1α. Stars indicate the species of interest. (a), Combined ITS, LSU and *tef*-1α (Coetzee et al. 2011), (b) Combined 28S, *tef*-1α, *rpb2*, TUB, gpd and actin-1 (Koch et al. 2017), (c) Phylogenetic tree based on IGS-1 sequences showing the relationship of *A. jezoensis* and *A. singula* relative to other other *Armillaria* species (Terashima et al 1998), (d) Strict consensus tree of 210 most parsimonious ITS trees showing the relationship *of A. hinnulea* and *A. aotearoa* within the northern hemisphere clade of *Armillaria* species (Coetzee et al. 2001), (e) Tree generate from ITS sequenced showing the relationship of species from Argentina relative to other *Armillaria* species (Pildain et al. 2009).

C

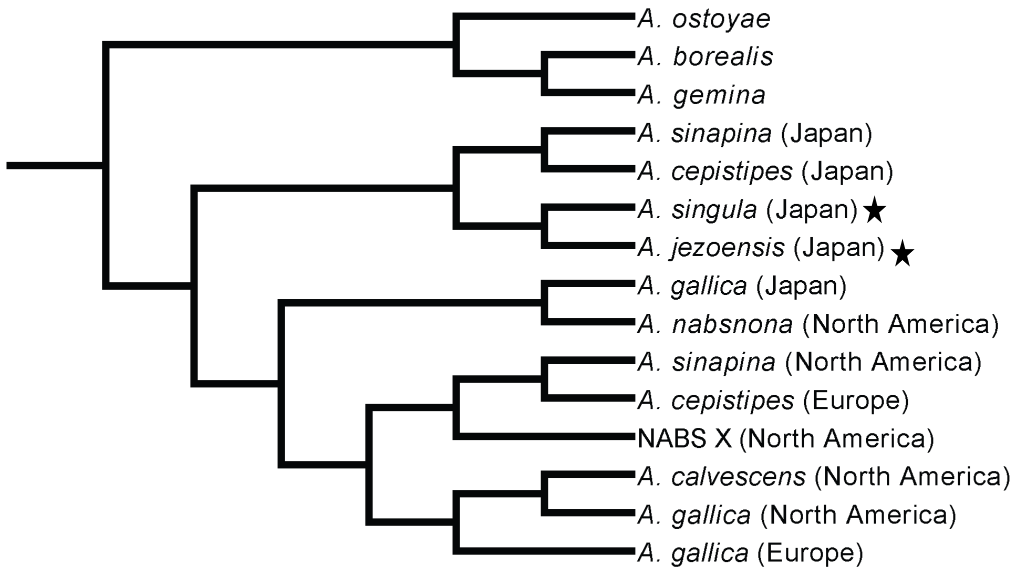
A. ostoyae
A. borealis
A. gemina
A. sinapina (Japan)
A. cepistipes (Japan)
A. singula (Japan) ★
A. jezoensis (Japan) ★
A. gallica (Japan)
A. nabsnona (North America)
A. sinapina (North America)
A. cepistipes (Europe)
NABS X (North America)
A. calvescens (North America)
A. gallica (North America)
A. gallica (Europe)

D

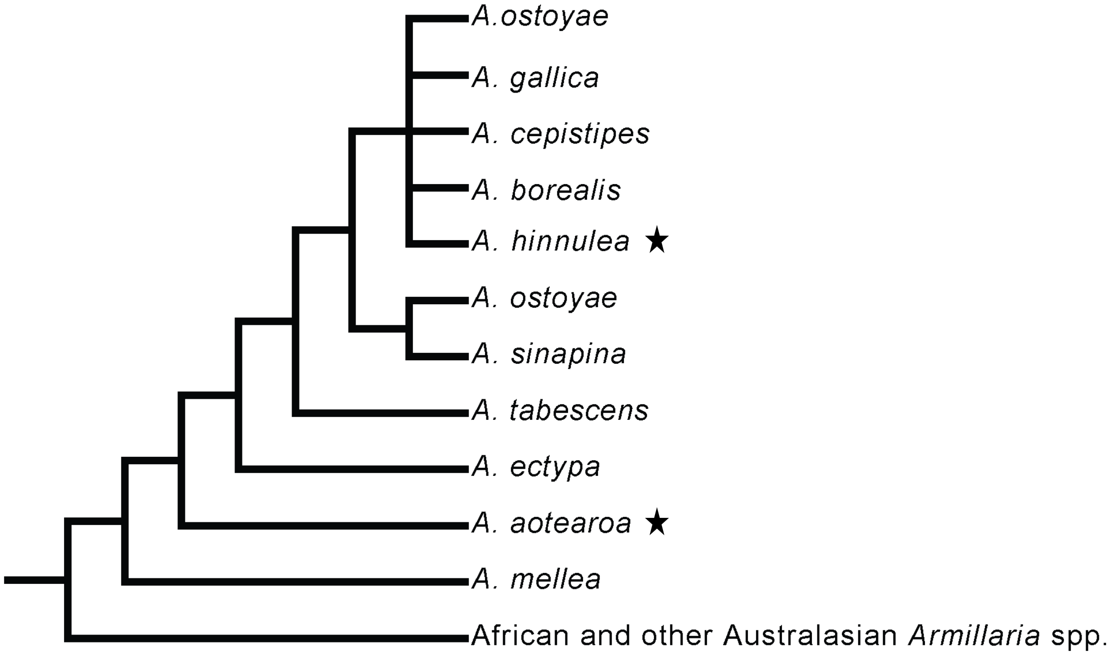
A.ostoyae
A. gallica
A. cepistipes
A. borealis
A. hinnulea ★
A. ostoyae
A. sinapina
A. tabescens
A. ectypa
A. aotearoa ★
A. mellea
African and other Australasian Armillaria spp.

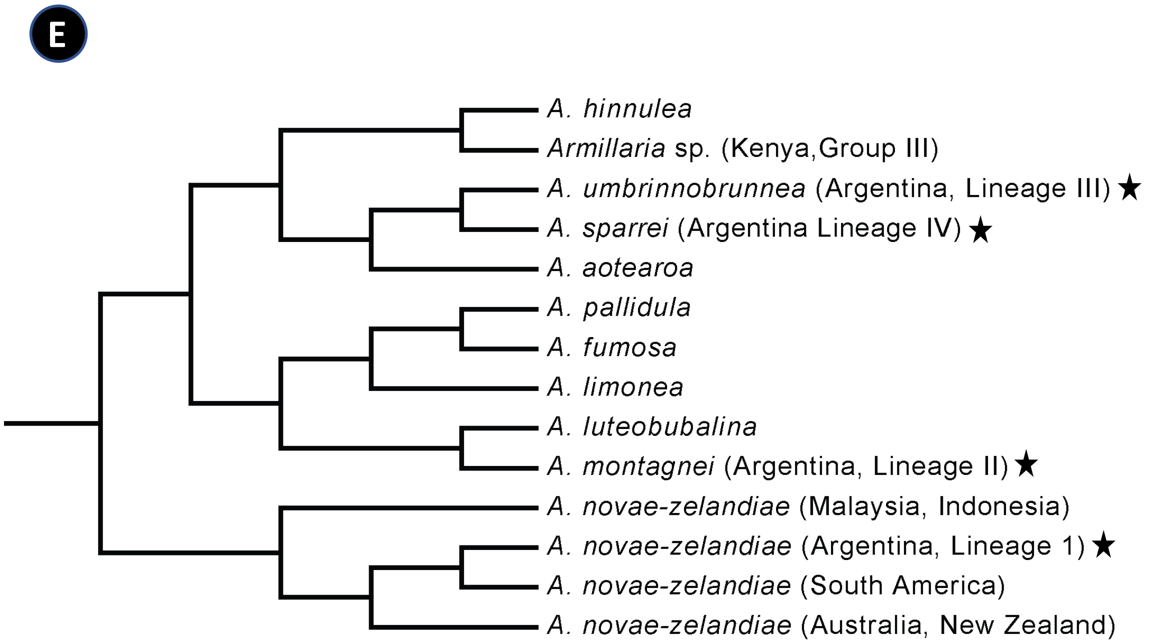
E
A. hinnulea
Armillaria sp. (Kenya,Group III)
A. umbrinnobrunnea (Argentina, Lineage III) ★
A. sparrei (Argentina Lineage IV) ★
A. aotearoa
A. pallidula
A. fumosa
A. limonea
A. luteobubalina
A. montagnei (Argentina, Lineage II) ★
A. novae-zelandiae (Malaysia, Indonesia)
A. novae-zelandiae (Argentina, Lineage 1) ★
A. novae-zelandiae (South America)
A. novae-zelandiae (Australia, New Zealand)
