# Supplementary material for: Armillaria Root-Rot Pathogens: Species Boundaries and Global Distribution
Source: Pathogens. 2018 Oct 24;7(4):83. doi: 10.3390/pathogens7040083 (PMC6313743; doi:10.3390/pathogens7040083)
Supplement: Supplementary file 1 [file pathogens-07-00083-s001.zip › pathogens-368392-supplementrary final/Table S1 Summary of genes and genomic regions used in Armillaria phylogenetics.docx]

**Table S1:** Summary of genes and genomic regions employed in phylogenetic studies of *Armillaria* species

| Gene or genomic region | Comments |
| --- | --- |
| IGS-1 | - The first region used in *Armillaria* phylogenetics - Does not provide sufficient resolution for discriminating some *Armillaria* species - 5S gene is inverted in African taxa, the region can therefore not be used for a global phylogenetic analysis - Intra-strain nucleotide heterogeneity present in some individuals |
| ITS | - Does not provide sufficient resolution for discriminating some *Armillaria* species - Intra-strain nucleotide heterogeneity present in some individuals |
| *tef*-1α | - In general, provides sufficient resolution to discriminate *Armillaria* species, it is now widely used in *Armillaria* phylogenetic studies - Some individuals, however, reported to have intra-strain heterozygous sites |
| *rpb2* | - Cannot discriminate between certain species, for example *A. calvescens* and *A. gallica* |
| β–tubulin | - Does not provide sufficient resolution to separate *Armillaria* species |
| SWAPP | - Phylogenetic analysis of a matrix in which these sequences were combined yielded a phylogenetic tree that resolved the phylogenetic history of the North American species - Has not been employed in other phylogenetic studies of *Armillaria* |
|  |  |
