# Supplementary material for: Armillaria Root-Rot Pathogens: Species Boundaries and Global Distribution
Source: Pathogens. 2018 Oct 24;7(4):83. doi: 10.3390/pathogens7040083 (PMC6313743; doi:10.3390/pathogens7040083)
Supplement: Supplementary file 1 [file pathogens-07-00083-s001.zip › pathogens-368392-supplementrary final/Table S3 Additional tef-1 DNA sequences .docx]

**Table S3:** Additional *tef*-1α DNA sequences from *Armillaria* species or taxa included in this study.

| **Biological Number** | **Species / Taxon** | **Culture numbers** | **Country of origin** | **GenBank number** | **Reference** |
| --- | --- | --- | --- | --- | --- |
| Arm00098 | *A. tabescens* | CMW31118, KK: 3480/16, Qin: 99022 | China | KM205280 | [25] |
| Arm00099 | CBS F | CMW31127, KK: 3355/1, Qin: 01107 | China | KM205286 | [25] |
| Arm00100 | CBS F | CMW31128, KK: 3424/1, Qin: 99102 | China | KM205287 | [25] |
| Arm00101 | CBS F | CMW31129, KK: 3405/1, Qin: 96060 | China | KM205288 | [25] |
| Arm00102 | CBS F | CMW31130, KK: 3426/1, Qin: 99107 | China | KM205289 | [25] |
| Arm00103 | CBS H | CMW31136, KK: 3419/1, Qin: 99012 | China | KM205293 | [25] |
| Arm00106 | CBS J | CMW31140, KK: 3154/1 | China | KM205296 | [25] |
| Arm00107 | CBS J | CMW31142, KK: 3333/1, Qin: 00101 | China | KM205297 | [25] |
| Arm00110 | CBS N | CMW31146, KK: 3365/3, Qin: 02068 | China | KM205300 | [25] |
| Arm00111 | CBS N | CMW31148, KK: 3363/3, Qin: 02066 | China | KM205301 | [25] |
| Arm00112 | CBS O | CMW31150, KK: 3369/2, Qin: 02072 | China | KM205302 | [25] |
| Arm00113 | CBS O | CMW31151, KK: 3369/13, Qin: 02072 | China | KM205303 | [25] |
| Arm00114 | CBS C | CMW31123, KK: 3428/8, Qin: 99110 | China | KM205284 | [25] |
| Arm00115 | CBS C | CMW31124, KK: 3409/2, Qin: 97047 | China | KM205285 | [25] |
| Arm00116 | Chinese Lineage 1 | HKAS 86615, 00126/2 | China | KT822384 | [6] |
| Arm00118 | Chinese Lineage 1 | HKAS 86622, 02071/2 | China | KT822390 | [6] |
| Arm00119 | Chinese Lineage 1 | HKAS 85519, Gt794 | China | KT822373 | [6] |

**Table S3 (continued)**

| **Biological Number** | **Species / Taxon** | **Culture numbers** | **Country of origin** | **GenBank number** | **Reference** |
| --- | --- | --- | --- | --- | --- |
| Arm00120 | Chinese Lineage 1 | HKAS 85527, Gt802 | China | KT822374 | [6] |
| Arm00121 | Chinese Lineage 1 | HKAS 85551, Gt826 | China | KT822382 | [6] |
| Arm00122 | Chinese Lineage 1 | HKAS 85575, Gt850 | China | KT822383 | [6] |
| Arm00123 | Chinese Lineage 1 | HKAS 85581, Gt856 | China | KT822392 | [6] |
| Arm00126 | Chinese Lineage 2 | HKAS 86551, Guo282 | China | KT822367 | [6] |
| Arm00127 | Chinese Lineage 2 | HKAS 86554, Guo322 | China | KT822366 | [6] |
| Arm00132 | Chinese Lineage 3 | HKAS 86614, 00125/2 | China | KT822391 | [6] |
| Arm00133 | Chinese Lineage 3 | HKAS 86548, Guo278 | China | KT822395 | [6] |
| Arm00138 | Chinese Lineage 4 | HKAS 86602, 00019/4 | China | KT822378 | [6] |
| Arm00139 | Chinese Lineage 4 | HKAS 86606, 00107/2 | China | KT822359 | [6] |
| Arm00141 | Chinese Lineage 4 | HKAS 86609, 00117/7 | China | KT822372 | [6] |
| Arm00147 | Chinese Lineage 4 | HKAS 85594, Gt869 | China | KT822377 | [6] |
| Arm00148 | Chinese Lineage 4 | HKAS 51692, Yang4881 | China | KT822415 | [6] |
| Arm00151 | Chinese Lineage 5 | HKAS 86576, 99109/6 | China | KT822360 | [6] |
| Arm00153 | Chinese Lineage 5 | HKAS 86578, 99115/10 | China | KT822365 | [6] |
| Arm00159 | Chinese Lineage 6 | HKAS 85572, Gt847 | China | KT822400 | [6] |
| Arm00160 | Chinese Lineage 6 | HKAS 86564, Guo341 | China | KT822405 | [6] |
| Arm00167 | Chinese Lineage 7 | HKAS 83361, Qin944 | China | KT822436 | [6] |

**Table S3 (continued)**

| **Biological Number** | **Species / Taxon** | **Culture numbers** | **Country of origin** | **GenBank number** | **Reference** |
| --- | --- | --- | --- | --- | --- |
| Arm00168 | Chinese Lineage 7 | HKAS 86541, Xhwang3394 | China | KT822435 | [6] |
| Arm00280 | Chinese Lineage 6 | HKAS 86558, A9 | Korea | KT822407 | [6] |
| Arm00231 | *A. ectypa* | TFM27105, Je-2 | Japan | AB558992 | [95] |
| Arm00243 | *A. mellea* | HKAS 86588, 83003/2 | Japan | KT822352 | [6] |
| Arm00266 | *A. tabescens* | MAFF420667, WD2607, TFM27084, 96-1-8 | Japan | AB510804 | [38] |
| Arm00277 | *A. mellea* | HKAS 86598, PFD84-103 | Kenya | KT822348 | [6] |
| Arm00314 | *A. novae-zelandiae* | CMW4722, G3.0.34.4 | New Zealand | DQ435652 | [26] |
| Arm00431 | *Armillaria* Zimbabwean Group III | CMW9954, P21 | Zimbabwe | DQ435620 | [26] |
| Arm00453 | *Armillaria* sp. | CMW4456, Z1 | Zimbabwe | DQ435617 | [26] |
| Arm00519 | *A. luteobubalina* | CMW4977, SA(6) | Australia | DQ435657 | [26] |
| Arm00606 | *A. mexicana* | MEX87, CM-CNRG 399 | Mexico | KR061314 | [28] |
| Arm00607 | *A. mexicana* | MEX88, CM-CNRG 365 | Mexico | KR061315 | [28] |
