# Supplementary material for: Armillaria Root-Rot Pathogens: Species Boundaries and Global Distribution
Source: Pathogens. 2018 Oct 24;7(4):83. doi: 10.3390/pathogens7040083 (PMC6313743; doi:10.3390/pathogens7040083)
Supplement: Supplementary file 1 [file pathogens-07-00083-s001.zip › pathogens-368392-supplementrary final/Table S4 Distribution of Armillaria species .docx]

**Table S4:** Distribution of *Armillaria* species and taxa from phylogenetic studies and their associated biological species designation.

| **Species or taxon** | **Location and biological species^1^** | | | | | | | | | | | |
| --- | --- | --- | --- | --- | --- | --- | --- | --- | --- | --- | --- | --- |
|  | **North America** | | |  | **East Asia** | | |  |  |  |  |  |
|  | **NAm (east)** | **NAm (west)** | **South NAm and Central Am** | **Europe** | **China** | **Japan** | **South Korea** | **Africa** | **South America** | **New Zealand** | **Australia** | **Other locations** |
| African *Armillaria* Clade B | |  |  |  |  |  |  | * |  |  |  |  |
| *A. affinis* |  |  | * |  |  |  |  |  |  |  |  |  |
| *A. altimontana* |  | NABS X |  |  |  |  |  |  |  |  |  |  |
| *A. aoteareo* |  |  |  |  |  |  |  |  |  | * |  |  |
| *A. borealis* (cf.) |  |  |  | EBS A | CBS M |  |  |  |  |  |  |  |
| *A. calvescens* | NABS III |  |  |  |  |  |  |  |  |  |  |  |
| *A. camerunensis* |  |  |  |  |  |  |  | * |  |  |  |  |
| *A. cepistipes* |  | NABS XI |  | EBS B | CBS F | NAG D | * |  |  |  |  |  |
| *A. fumosa* |  |  |  |  |  |  |  |  |  |  | * |  |
| *A. fuscipes* |  |  |  |  |  |  |  | * |  |  |  |  |
| *A. gallica* | NABS VII | NABS VII | * | EBS E |  | NAG A | * |  |  |  |  | Hawaii |

**Table S4 (continued)**

| **Species or taxon** | **Location and biological species^1^** | | | | | | | | | | | |
| --- | --- | --- | --- | --- | --- | --- | --- | --- | --- | --- | --- | --- |
|  | **North America** | | |  | **East Asia** | | |  |  |  |  |  |
|  | **NAm (east)** | **NAm (west)** | **South NAm and Central Am** | **Europe** | **China** | **Japan** | **South Korea** | **Africa** | **South America** | **New Zealand** | **Australia** | **Other locations** |
| *A. gemina* | NABS II |  |  |  |  |  |  |  |  |  |  |  |
| *A. hinnulea* |  |  |  |  |  |  |  |  |  | * | * |  |
| *A. jezoensis* |  |  |  |  |  | H |  |  |  |  |  |  |
| *A. limonea* |  |  |  |  |  |  |  |  |  | * |  |  |
| *A. luteobubalina* |  |  |  |  |  |  |  |  |  |  | * | * |
| *A. mellea* | NABS VI | NABS VI | * | EBS D | CBS K |  |  |  |  |  |  | Iran |
| *A. mellea* (homothallic) |  |  |  |  | CBS G | NAG Am |  |  |  |  |  |  |
| *A. mexicana* |  |  | Mexico |  |  |  |  |  |  |  |  |  |
| *A. montagnei* |  |  |  |  |  |  |  |  | * |  |  |  |
| *A. nabsnona* |  | NABS IX |  |  |  | NAG B | * |  |  |  |  |  |
| *A. novae-zelandiae* |  |  |  |  |  | * |  |  | * | * | * | Papua New Guinea, Indonesia, Malaysia and Amami-Oshima |

**Table S4 (continued)**

| **Species or taxon** | **Location and biological species^1^** | | | | | | | | | | | |
| --- | --- | --- | --- | --- | --- | --- | --- | --- | --- | --- | --- | --- |
|  | **North America** | | |  | **East Asia** | | |  |  |  |  |  |
|  | **NAm (east)** | **NAm (west)** | **South NAm and Central Am** | **Europe** | **China** | **Japan** | **South Korea** | **Africa** | **South America** | **New Zealand** | **Australia** | **Other locations** |
| *A. ostoyae* (*solidipes*) | NABS I | NABS I |  | EBS C | CBS D | NAG C | * |  |  |  |  |  |
| *A. pallidula* |  |  |  |  |  |  |  |  |  |  | * |  |
| *A. paulensis* |  |  |  |  |  |  |  |  | * |  |  |  |
| *A. puggaria* |  |  |  |  |  |  |  |  |  |  |  | Guadeloupe |
| *A. sinapina* | NABS V | NABS V |  |  | CBS A | F |  |  |  |  |  |  |
| *A. singula* |  |  |  |  |  | G |  |  |  |  |  |  |
| *Armillaria* sp. |  |  |  |  |  |  |  | Kenya |  |  |  |  |
| *A. sparrei* |  |  |  |  |  |  |  |  | * |  |  |  |
| *A. umbrinnobrunnea* |  |  |  |  |  |  |  |  | * |  |  |  |
| Chinese Lineage 1 |  |  |  |  | CBS L |  |  |  |  |  |  |  |
| Chinese Lineage 1 |  |  |  |  | CBS N |  |  |  |  |  |  |  |
| Chinese Lineage 2 |  |  |  |  | CBS O |  |  |  |  |  |  |  |
| Chinese Lineage 3 |  |  |  |  | CBS L |  |  |  |  |  |  |  |
| Chinese Lineage 4 |  |  |  |  | CBS M |  |  |  |  |  |  |  |

**Table S4 (continued)**

| **Species or taxon** | **Location and biological species^1^** | | | | | | | | | | | |
| --- | --- | --- | --- | --- | --- | --- | --- | --- | --- | --- | --- | --- |
|  | **North America** | | |  | **East Asia** | | |  |  |  |  |  |
|  | **NAm (east)** | **NAm (west)** | **South NAm and Central Am** | **Europe** | **China** | **Japan** | **South Korea** | **Africa** | **South America** | **New Zealand** | **Australia** | **Other locations** |
| Chinese Lineage 4 |  |  |  |  | CBS H |  |  |  |  |  |  |  |
| Chinese Lineage 4 |  |  |  |  | CBS J |  |  |  |  |  |  |  |
| Chinese Lineage 5 |  |  |  |  | CBS C |  |  |  |  |  |  |  |
| Chinese Lineage 6 |  |  |  |  | CBS B |  |  |  |  |  |  |  |
| *D. ectypa* |  |  |  | * |  | * |  |  |  |  |  |  |
| *D. tabescens* | * | * | * | * | CBS I | T | * |  |  |  |  |  |
| Japanese Lineage NAG E |  |  |  |  |  | NAG E |  |  |  |  |  |  |
| Zimbabwean Group II |  |  |  |  |  |  |  | Zimbabwe |  |  |  |  |
| Zimbabwean Group III |  |  |  |  |  |  |  | Zimbabwe |  |  |  |  |
| Zimbabwean Group IV |  |  |  |  |  |  |  | Zimbabwe |  |  |  |  |
| Zimbabwean Group V |  |  |  |  |  |  |  | Zimbabwe |  |  |  |  |
| *A. mellea* ssp*. africana* |  |  |  |  |  |  |  | Kenya |  |  |  |  |
| *A. mellea* spp*. nipponica* |  |  |  |  |  | * |  |  |  |  |  |  |
| BPS 1 |  |  |  |  |  |  |  |  |  |  |  | Bhutan |

**^1^**Location and biological species updated from [6,25,29,100,108,128]. Abbreviations: NAm = North America, Am = America, NABS = North American biological species, EBS = European biological species, CBS = Chinese biological species, NAG = Japanese biological species, BPS = Bhutanese phylogenetic species., Letters in columns indicate biological species designation. * indicates the presence of the species in a region, but for which an equivalent biological species name is not available.

References

1. Baumgartner, K.; Coetzee, M.P.A.; Hoffmeister, D. Secrets of the subterranean pathosystem of *Armillaria*. *Mol. Plant Pathol.* **2011**, *12*, 515–534, doi:10.1111/j.1364-3703.2010.00693.x.
